# Quantitative Comparative Proteomics Reveal Biomarkers for Dengue Disease Severity

**DOI:** 10.3389/fmicb.2019.02836

**Published:** 2019-12-10

**Authors:** Lifen Han, Xiulan Ao, Shujin Lin, Shengcan Guan, Lin Zheng, Xiao Han, Hanhui Ye

**Affiliations:** ^1^The United Innovation of Mengchao Hepatobiliary Technology Key Laboratory of Fujian Province, Mengchao Hepatobiliary Hospital of Fujian Medical University, Fuzhou, China; ^2^College of Biological Science and Engineering, Fuzhou University, Fuzhou, China

**Keywords:** proteomomics, dengue fever, dengue hemorrhagic fever, biomarker, TMT

## Abstract

Dengue fever (DF) could develop into dengue haemorrhagic fever (DHF) with increased mortality rate. Since the clinical characteristics and pathogen are same in DF and DHF. It’s important to identify different molecular biomarkers to predict DHF patients from DF. We conducted a clinical plasma proteomics study using quantification (TMT)-based quantitative proteomics methodology to found the differential expressed protein in DF patients before they developed into DHF. In total 441 proteins were identified up or down regulated. There proteins are enriched in diverse biological processes such as proteasome pathway, Alanine, aspartate, and glutamate metabolism and arginine biosynthesis. Several proteins such as PLAT, LAMB2, and F9 were upregulated in only DF patients which developed into DHF cases, not in DF, compared with healthy-control. In another way, FGL1, MFAP4, GLUL, and VCAM1 were upregulated in both DHF and DF cases compare with healthy-control. RT-PCR and ELISA were used to validate these upregulated gene expression and protein level in 54 individuals. Results displayed the same pattern as proteomics analysis. All including PLAT, LAMB2, F9, VCAM1, FGL1, MFAP4, and GLUL could be considered as potential markers of predicting DHF since the levels of these proteins vary between DF and DHF. These new founding identified potential molecular biomarkers for future development in precision prediction of DHF in DF patients.

## Introduction

Dengue fever (DF) is an acute virus infectious disease mainly prevalent in tropical and subtropical zone. In severe cases, it could develop into dengue haemorrhagic fever (DHF), resulting in a rapid deterioration of conditions and a high mortality rate. The clinical characteristics of DHF are similar as DF at the onset of disease (Calderon-Pelaez et al., 2019; Halstead, 2019). Among the pool of dengue patients, predicting who will develop DHF is the key challenge.

The clinical diagnosis of DF mainly includes serological examination, NS1 antigen detection, virus isolation, and viral nucleic acid detection assays. Based on these methods, studies developed many potential molecular approaches to detect DHF from DF patients. Vasanthapuram et al. conducted routine serological surveillance on suspected patients with DF in New Delhi, India (Vasanthapuram et al., 2019). Researchers found that the levels of Interleukins (ILs) and Interferon-γ (IFN-γ) are significantly increased in the serum of severe patients with dengue virus infection (Bozza et al., 2008). Furuta et al. (2012) found that the IL-17 levels in serum from DHF patients were significantly higher than those in DF patients or in healthy subjects. Non-Structural-1 (NS1) titers could be used to predict the occurrence of DHF (Huhtamo et al., 2010; Laurent et al., 2010; Singh et al., 2010). However, it is still no clinical diagnosis for DHF by the above methods.

Proteomics has the characteristics of high throughput and the ability to detect low abundance serum proteins. Serum proteomics analysis could be used as an assistive technology to explore biomarkers and study the pathogenesis of diseases. For example, Avent (2013) applied proteomics technology to study the biomarkers for Down syndrome (DS) and identified the protein markers differentiating the DS group from the control group. The diagnostic accuracy of these markers is as high as 96%. Proteomics has been increasingly applied to the screening of biomarkers (Bonotti et al., 2017; Frantzi and Vlahou, 2017; Gajbhiye et al., 2017). Recently, potential biomarkers related to DF and DHF, including antithrombin III, alpha-2 macroglobulin, serotransferrin, and vitronectin, were identified by proteomics with non-quantitative method (Nhi et al., 2016). However, the quantitative analysis of DF and DHF serum proteomics is still unclear.

This study is designed to identify stable and specific biomarkers by quantitative proteomics analysis from the serum of DF, DHF patients and health person. A series of proteins were validated by Western blot and ELISA s as potential biomarkers for early detection of DHF.

## Materials and Methods

### Patients and Sample Collection

This study was approved by the Ethics Committee of the Mengchao Hepatobiliary Hospital of Fujian Medical University. The study population included hospitalized patients first identified as DF in Mengchao Hepatobiliary Hospital of Fujian Medical university from June 2016 to October 2016. The patient conformed to the clinical manifestations of DF and had a decrease in white blood cells and platelets. The single serum was positive for dengue virus specific IgM antibody and antigen NS1. Blood samples were drawn from participants at the time of dengue diagnosis with the onset of disease being less than 3 days. Based on the final outcome during hospital stay, these patients were classified as those with DHF or DF. The DHF cases developed thrombocytopenia (<50 × 10^9^/L) and other severe manifestations after the collection of blood samples. The DF group has no severe manifestations. Healthy controls in this work were age and gender matched. There were 18 samples in each group, and six samples were labeled with TMT for a mixed pool.

### Protein Preparation

A 10 mL volume of venous blood was obtained from fasted participants in both the control group and the experimental group and was concentrated. A ProteoExtract^®^ Albumin/IgG Removal Kit (Merck, United States) was used to remove these two kinds of proteins with a high abundance in serum. The operation was carried out according to the instructions of the kit. The serum was removed from the −80°C freezer. Extraction buffer (100 mM Tris–HCl (Amresco, United States), 100 mM dithiothreitol (DTT) (Sigma, United States), and 2% SDS (Amresco, United States), pH 7.6) were added. Then, the samples were heated (95°C, 5 min) and centrifuged (20,000 × *g*, 5 min) at room temperature, and the supernatant was collected.

### Protein Digestion

After quantification by the Bradford (Bio-Rad, United States) method, protein digestion was performed according to the cold acetone precipitation procedure. Briefly, 100 μg of protein from each sample was reduced. DTT was added to the protein extract to 20 mM. The extract was then kept at 37°C for 1 h. Iodoacetamide (IAM) (Sigma, United States) was then added to a final concentration of 100 mM. The mixture was then left for 30 min at room temperature in the dark. More than five volumes of acetone (Thermo Scientific, United States) precooled at −20°C was added. The mixture was left overnight at −20°C and centrifuged at 20,000 × *g* for 20 min. The supernatant was removed, and the resulting pellet was air-dried to remove the residual acetone and dissolved in 50 mM TEAB (Sigma, United States). Finally, trypsin (Promega, United States) was added at a 1:100 enzyme: substrate ratio. The mixture was incubated overnight at 37°C, and the resulting peptide was collected.

### TMT Labeling

TMT six-plex Label Reagent Set (Thermo, United States) was used to label the samples. The labeling reagent was placed at room temperature for enough time. Acrylonitrile ACN (Fisher Scientific, United States) was added to vial of each labeling reagent and placed at room temperature for 5 min, during which the shaking was even. The liquids in each vial were added to the protein solution containing the same mass protein, and then blended. The liquids were placed at room temperature for 2 h. Then hydroxylamine (Sigma, United States) was added to each Ep tube to terminate the reaction. Samples of equal volume are drawn from each Ep tube to the new Ep tube.

### High pH Reversed-Phase Separation

The mixed sample was thoroughly dissolved using 100 μL of a solution containing 2% ACN (Fisher Scientific, United States) and 0.1% Fructosamine (FA) (Fisher Scientific, United States), and peptides were separated using an HPLC system. The HPLC conditions were as follows: Type of chromatographic column: TechMate C18-ST, 5 μm, 120 Å, 4.6 × 250 mm (Agilent, United States); Injection volume: 20 μL with 5 continuous injections; Flow rate: 600 μL/min; Mobile phase A: 98% H_2_O (Fisher Scientific, United States), 2% ACN and 5 mM NH_4_HCO_3_ (Fluka, United States); Mobile phase B: 10% H_2_O, 90% ACN and 5 mM NH_4_HCO_3_; Elution gradient: 0–6 min (5% B), 6–40 min (5–50% B), 40–43 min (50–90% B), 43–46 min (90% B), 46–46.1 min (90–5% B), and 46.1–50 min (5% B). A total of 48 fractions were collected. The collected fractions were combined into 12 fractions.

### Low pH Nano-Liquid Chromatography Tandem MS (Nano-LC-MS/MS) Analysis

Next, the peptides were analyzed with a 90-min gradient nano-LC-MS/MS system equipped with an AB SCIEX TripleTOF 6600. The autosampler was used for loading, with a single injection volume of 4 μL and an injection flow rate of 4 μL/min. The nano-LC conditions were as follows. Mobile phase C: 95% H_2_O (Fisher Scientific, United States), 0.1% FA (Fisher Scientific, United States) and 5% DMSO (Sigma, United States); Mobile phase D: 95% ACN (Fisher Scientific, United States), 0.1% FA and 5% DMSO; Trap column: Nano cHiPLC trap column (200 μm × 0.5 mm, ChromXP C18-CL 3 μm, 120 Å) (SCIEX, United States); Analytical column: Nano cHiPLC column (75 μm × 15 cm, ChromXP C18-CL 3 μm, 120 Å) (SCIEX, United States); Flow rate: 300 nL/min; Elution gradient: 0–0.5 min (5–7% D), 0.5–60 min (7–28% D), 60–72 min (28–30% D), 72–77 min (30–50% D), 77–77.1 min (50–90% D), 77.1–82 min (90–10% D), 82–82.1 min (10–5% D), and 82.1–90 min (5% D).

The eluted fraction was passed directly into the mass spectrometer via a nano-ESI ion source. Mass spectral data were acquired based on the high-resolution TripleTOF 6600 mass spectrometry system. The TOF MS mode was used. The TOF mass analyzer has a mass-to-charge ratio range of 350–1500 m/z. The cumulative time was 0.25 s. The positive ion mode was selected. The first 40 ions in the cycle were selected for MS/MS acquisition. The cumulative time to acquire each MS/MS spectrum was 0.05 s. The parent ions with charge numbers of 2–4 were selected for MS/MS analysis with the following conditions: Voltage of ion source: 2.4 kV; GS1: 5 psi; CUR: 35 psi; DP: 80 V; and CES: 5.

### TMT Data Analysis

All raw files were searched using MaxQuant version 1.6.1.0 against the *Homo sapiens* database obtained from UniProt^1^. The parameter settings for the library search were as follows: Sample Type: Identification; Cys Alkylation: Iodoacetamide; Digestion: Trypsin; Instrument: Triple TOF 6600; Special Factors: None; Species: *Homo sapiens*; ID Focus: Biological Modifications; Search Effort: Thorough ID; Detected Protein Threshold (Unused ProtScore): 0.05% (10%); FDR: YES; and Bias Correction: YES. The raw data accompanying this study have been deposited into the iProX with the identifier IPX0001691000.

All sample quantitative data were statistically analyzed using Persus version 1.6.1.1. Proteins were classified by Gene Ontology (GO) and the KEGG DAVID tool to explain biological processes, molecular functions, and cellular components.

### RT-qPCR

The whole blood from patients were used for detection. RNA was extracted by Trizol reagent (Life Technologies, United States). *ACTIN* was used as the reference gene. The cDNA was then reverse-transcribed using the First Strand cDNA Synthesis Kit (TOYOBO, Japan). Then, the cDNA samples were subjected to real-time quantitative PCR using a TransStart Top Green qPCR SuperMix (TransGen Biotech, China) on a GeneAmp PCR System 2400 Thermal Cycler (PerkinElmer, Wellesley, MA, United States).

### Western Blot and ELISA Analysis

The protein from the whole blood of patients were used for detection. A total of 20 μg of protein was used per sample. Proteins were first separated via 12% SDS-PAGE (Solarbio, China) and were then transferred to PVDF (Merck, United States) membranes. The PVDF membranes were then incubated with primary antibodies (Abcam, United Kingdom) corresponding to the protein of interest. Visualization was performed after incubation with the secondary antibody (Abcam, United Kingdom). Bands were visualized using ECL (Abcam, United Kingdom). Quantitative analysis of the Western blot results was performed using ImageJ analysis software^2^. GAPDH was used as the reference gene. For anticoagulation treatment, 3.8% sodium citrate was added to the collected peripheral blood of the patients. After 15 min, the plasma was centrifuged at 1000 *g* for 15 min at 4°C. The supernatant was taken to determine the concentration, and then used for ELISA detection (Abcam, United Kingdom). The concentrations of protein were quantified using ELISA according to the manufacturer’s protocol. In brief, a 96-well plate was added with sample, and incubated 2 h at 37°C. Then, remove the liquid of each well, added 100 μl HRP-avidin, and incubate for 1 h at 37°C. After washing, 90 μl of TMB substrate was added for 30 min. Finally, 50 μl of stop solution was added. The plate was read at 450 nm on an ELISA plate reader (Tecan) within 5 min. The data was exported to Excel, three replicates were averaged, and the standard dilutions were fit to a linear curve.

### Statistical Analysis

Data are expressed as the means ± SDs. Student *T* test were carried out to compare the gene and protein level. A *p*-value was used to indicate statistical significance. ^∗^*p* < 0.05, ^∗∗^*P* < 0.01, and ^∗∗∗^*P* < 0.001.

## Results

### Workflow

**FIGURE 1:**
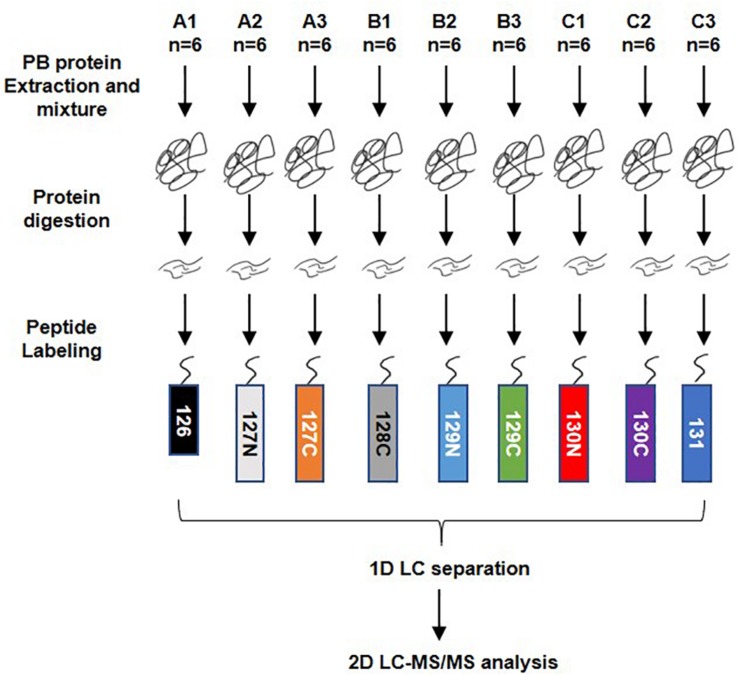
Workflow of TMT quantitatively proteomics analysis. A total of 54 serum samples from dengue haemorrhagic fever (DHF), Dengue fever (DF), and healthy groups were collected, numbered A, B, and C, with 18 in each group. After protein extraction, the serum proteins of every six people in the group were mixed into a mixed pool for TMT labeling. The order of labeling is: A1-126, A2-127N, A3-127C, B1-128C, B2-129N, B3-129C, C1-130N, C2-130C, C3-131. When the labeling was completed, all the proteins were mixed and rotated to dry. The 1D LC separation – high-pH reverse-phase separation was performed after resolving. The separated components are combined and evaporated to dryness and then re-dissolved. LC-MS/MS analysis was performed after that.

A total of 54 serum samples from the DHF (18), DF (18), and healthy groups (18) were collected; the groups were named A, B, and C, respectively. The DHF group developed to a condition with decreased platelets (<50 × 10^9^/L) and other severe manifestations after we collected blood sample. The DF group has no severe manifestations. After protein extraction, the serum protein of every subset of six people in each group was mixed into a pool for TMT labeling. The order of labeling was as follows: A1-126, A2-127N, A3-127C, B1-128C, B2-129N, B3-129C, C1-130N, C2-130C, and C3-131. LC-MS/MS analysis was performed after high pH reversed-phase separation (Figure 1).

### Clinical Indicators of the Patients

The median ages of the three groups (A, B, and C) were 34 ±6.5, 34 ±8, and 34 ±7.2 years old, respectively, and the ratio of male to female in each group was 1:1. The virus classification for group A was I (17 people) and III (1 person) and for group B was I (18 people). The mean disease course durations were 4.61 ±2.09 days for group A and 3.61 ±2.73 days for group B. In groups A, B, and C, the number of patients positive for IgG in serum was 3, 1, and 0; the number positive for IgM in serum was 3, 2, and 0; and the number positive for NS1 antigen was 18, 18, and 0, respectively. The mean blood platelet counts in groups A and B were 37 ±3.1, 142 ±5.2, respectively (× 10^9^ as a unit of measurement) (Table 1).

**TABLE 1:** Clinical indicators of the patients.

	**A (DHF)**	**B (DF)**	**C (healthy)**
Ages, mean ±SD	34 ±6.5	34 ±8	34 ±7.2
Male (%)	50% (9/18)	50% (9/18)	50% (9/18)
Serotype	I (17), III (1)	I (18)	–
Previous history	+(1), −(17)	+(1), −(17)	–
Disease course, days	4.61 ±2.09	3.61 ±2.73	–
IgG	+ (3), −(15)	+(1), −(17)	–
IgM	+(3), −(15)	+(2), −(16)	–
NS1	+(18), −(0)	+(18), −(0)	–
blood platelet (10^9^/L)	37 ±3.1	142 ±5.2	230 + 10.1

*Group A, dengue haemorrhagic fever (DHF); B, dengue fever (DF); C, healthy.*

### Cluster Analysis of Differentially Expressed Proteins

**FIGURE 2:**
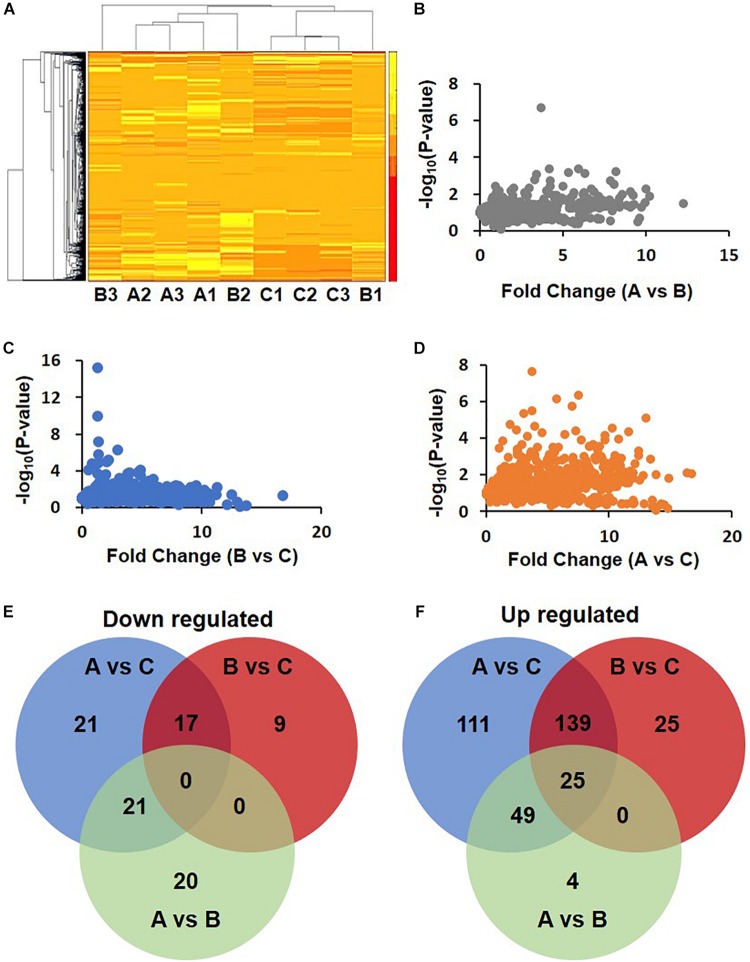
Differential expression proteins in DHF, DF compared with health person. **(A)** Clustering of proteomics data based on differential proteins. Note that the data in Group B show differences. B2 was clustered with A1, A2, and A3, while B1, C1, C2, and C3 were clustered together. **(B–D)** Volcano map of A/B, B/C, and A/C. **(E,F)** Venn diagram of down-regulated and up-regulated proteins.

Cluster analysis of these differentially expressed proteins was performed. The heat map showed that the upregulated and downregulated proteins formed three different clusters (Figure 2A). The difference between A and B was the least (Figure 2B). Slightly more proteins were differentially expressed between B and C (Figure 2C). The number proteins differentially expressed between A and C was the biggest (Figure 2D).

### Relative Quantification of the Serum Proteome in the Three Groups

The MaxQuant data were analyzed by Perseus 1.6. Using a fold change of 1.5 as a cutoff, 441 different proteins were identified in total. Among these proteins, 88 were downregulated. The number of downregulated proteins overlapping between groups A vs. C and B vs. C was 17 but was 21 between groups A vs. C and A vs. B (Figure 2E). In another way, 353 proteins were upregulated. The number of these differentially expressed proteins in groups A vs. C, B vs. C, and A vs. B were 324, 189, and 78, respectively. The number of proteins overlapping between groups A vs. C and B vs. C was 139. The upregulated proteins just in groups A vs. C and B vs. C are, 111 and 25, respectively (Figure 2F).

### Differentially Expressed Proteins Associated With DF and DHF

Gene Ontology functional analysis was used to classify the cellular components of these differentially expressed proteins. The differentially expressed proteins mainly existed as extracellular (Supplementary Figures S1A–D). In terms of the biological processes, the results of GO analysis showed that the upregulated proteins in group A vs. C were associated with cellular process (13%), single-organism process (12%), biological regulation (11%), response to stimulus (10%), and metabolic process (10%) (Supplementary Figure S2A). The downregulated proteins in group A vs. C were involved in single-organism process (10%) (Supplementary Figure S2B). The upregulated proteins in group B vs. C were associated with cellular process (13%), single-organism process (12%), biological regulation (11%), response to stimulus (10%), and metabolic process (10%) (Supplementary Figure S3A). The downregulated proteins in group B vs. C were involved in biological regulation (11%), single-organism process (10%), and cellular process (10%) (Supplementary Figure S3B).

### KEGG Pathway Analysis of the Differentially Expressed Proteins

**FIGURE 3:**
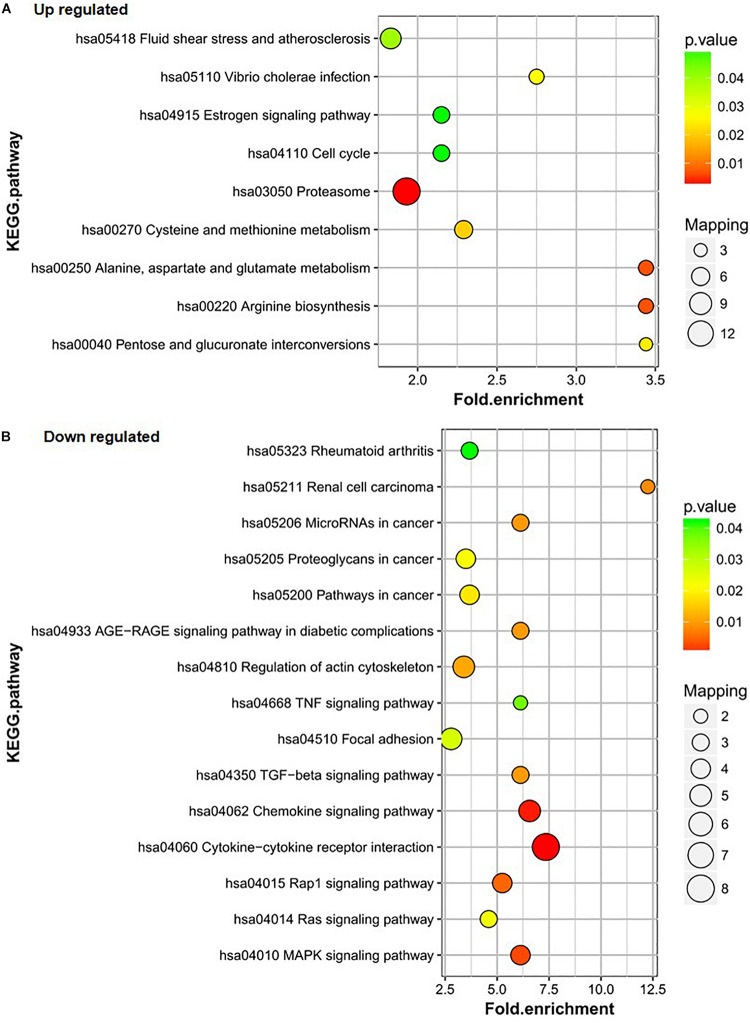
KEGG pathway analysis of the differentially expressed proteins of A/C. **(A)** Up-regulated proteins of A/C are enriched in diverse KEGG pathways. **(B)** The most significantly enriched pathways corresponding to the down-regulated proteins of A/C.

KEGG was used to further analyze the differentially expressed proteins in the healthy group (group C), DF group (group B), and DHF group (group A). The results showed that the upregulated proteins in group A vs. C were mainly involved in the processes of fluid shear stress and atherosclerosis; *Vibrio cholera* infection; estrogenic signaling pathway; cell cycle; proteasome; cysteine and methionine metabolism; alanine, aspartate, and glutamate metabolism; arginine biosynthesis; and pentose and glucoronate interconversions. The alanine, aspartate and glutamate metabolism and arginine biosynthesis pathways also changed significantly (Figure 3A). The three pathways in which downregulated proteins in group A vs. C were the most enriched were cytokine-cytokine receptor interaction, chemokine signaling pathway, and Mitogen-activated protein kinase (MAPK) signaling pathway; the number of proteins downregulated in group A vs. C in these pathways was 8, 4, and 4, respectively (Figure 3B).

**FIGURE 4:**
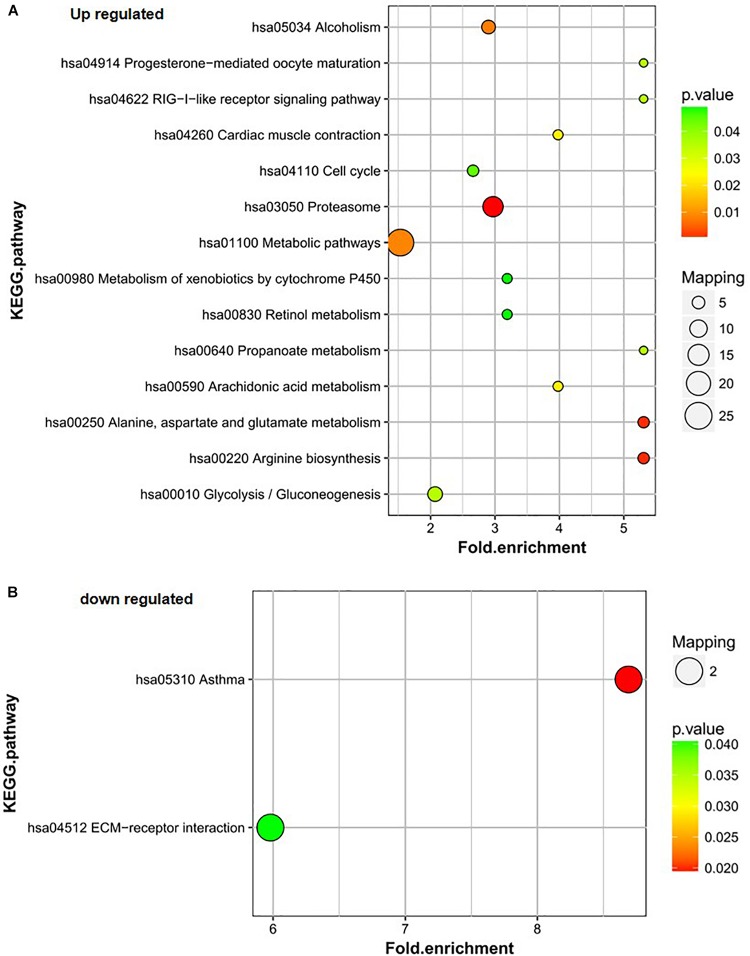
KEGG pathway analysis of the differentially expressed proteins of B/C. **(A)** Up-regulated proteins of B/C are enriched in diverse KEGG pathways. **(B)** The most significantly enriched pathways corresponding to the down-regulated proteins of B/C.

Proteins upregulated in group B vs. C mainly participated in the proteasome; alanine, aspartate, and glutamate metabolism; arginine biosynthesis; metabolic pathways; and alcoholism pathways (Figure 4A). The proteasome; alanine, aspartate, and glutamate metabolism; and arginine biosynthesis pathways were also the most significantly enriched pathways for the differentially expressed proteins. The downregulated proteins in group B vs. C were involved in only two pathways – asthma and ECM-receptor interaction. The asthma pathway was the main pathway. Only two proteins were involved in each pathway (Figure 4B).

### Validation of Differentially Expressed Proteins

**FIGURE 5:**
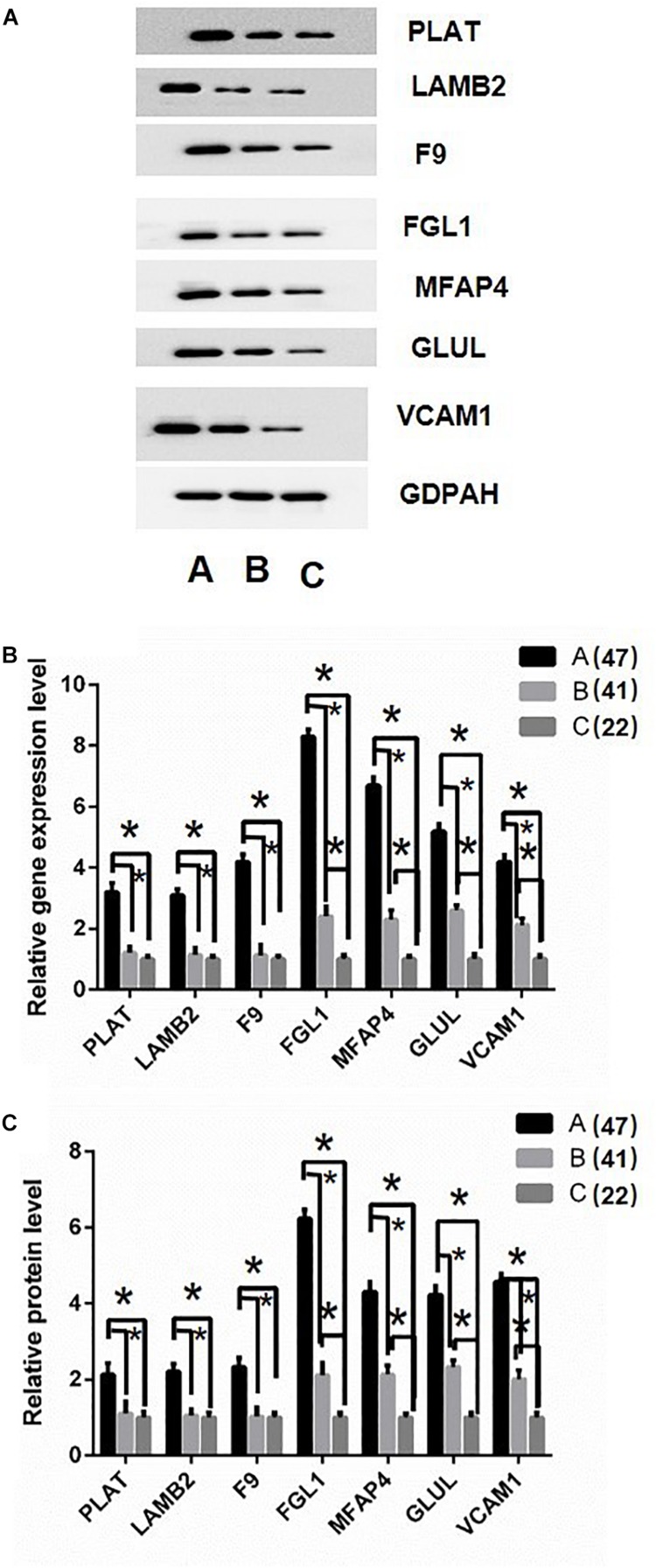
Validation of differentially expressed proteins. **(A)** western blot analysis of iTRAQ samples using GADPH as internal reference. **(B)** RT-qPCR analysis of genes in individual patients’ serum, DF, and DHF samples were collected when no severe manifestations detected, after detection, the DHF group has reduced platelets less than 50 × 10^9^/L. **(C)** Fold change of protein levels using ELISA analysis using the individual sample in RT-qPCR assay. The sample size were 47, 41, and 22, respectively. ^∗^*p* < 0.05. The data are shown as the mean values ± SD.

Based on the above analysis results, the key proteins were selected for verification. Firstly, protein just un-regulated in A (DHF) group including Plasminogen activator (PLAT), Laminin subunit beta 2 (LAMB2), and Coagulation factor IX (F9) were determined in sample pools by western blotting. Results displayed the same tendency as proteomics analysis (Figure 5A). Then, RT-PCR and ELISA were used to detect the RNA and protein level in all individual patients. Results indicated that PLAT, LAMB2, and F9 only increased gene and protein expression in A (DHF) group, but not in B (DF), compared with C (health) (Figure 5B).

Second, Fibrinogen-like protein 1 (FGL1), Microfibril-associated glycoprotein 4 (MFAP4), Glutamate-ammonia ligase (GLUL), and Vascular cell adhesion protein 1 (VCAM1) were selected for validation as they gradually up-regulated in A (DHF) and B (DF) groups. Western blotting results showed similar tendency as proteomics analysis in sample pools (Figure 5A). In individual patients, these gene expression were gradually induced from C (health), B (DF) to A (DHF). The changes of protein level showed the same pattern as gene expression.

## Discussion

The clinical symptoms of DF vary, which makes the diagnosis of DF difficult. Severe complications may occur in some DF patients, leading to DHF. The early stage of severe DF has all the typical manifestations of DF, but it suddenly worsens 3–5 days after the onset of the disease. New methods for the diagnosis, classification, and prediction of severe DF are essential. Some results have been obtained. Studies have shown that IL-10 (Tauseef et al., 2016), NO (Tun-Linn et al., 2015), sICAM-1 (Conroy et al., 2015) and other cytokines and proteins play a role in DF, DHF, and DSS and may be predictors of DF, DHF, and DSS. However, clinical studies of DF often show conflicting results. The results of the study by Conroy et al. (2015) on IL-10 in patients with DF are consistent with those of the study in Sri Lanka but different from the results reported in Venezuela. Tun-Linn et al. (2015) studied the bioavailability of NO in blood vessels of DHF patients in Singapore and found that significant increases in NO can be used as a predictor of DHF. However, the results of Trairatvorakul et al. (2005) in Thailand contrast with those findings – the serum NO level in DF patients was significantly lower than that in normal controls. In predicting DHF, the levels of vascular endothelial growth factor (VEGF) or its receptors were also inconsistent with those found in the study in Thailand (Conroy et al., 2015).

In this study, serum samples from the normal control group, DF group, and DHF group were analyzed by LC-MS/MS with TMT labeling, and the differentially expressed proteins between different groups were analyzed qualitatively and quantitatively. While, there are limitations in our experimental groups such as sample size are small, and not using non-dengue or other febrile illness group to be used as control. In addition, we utilized the WHO classification of 1997 for DF and DHF. Nowadays, the original classification is being used which categorizes dengue into dengue without warning signs, dengue with warning signs and severe dengue. Most of the DHF cases (grade I and grade II) fell into the group of dengue with warning signs. The current study could be only suggesting potential differential proteins between DF and DHF according to WHO classification in 1997.

In this study, we noted several proteins. The WB, RT-qPCR and ELISA results for these proteins were consistent with those of mass spectrometry. Four proteins VCAM1, FGL1, MFAP4, and GLUL were significantly upregulated in DF patients and very significantly upregulated in DHF patients compared with their expression in the healthy control group; thus, these proteins may be biomarkers for distinguishing DF from DHF. The expression of the PLAT, LAMB2 and F9 did not change significantly in DF patients compared with that in healthy controls but increased significantly in DHF individuals, suggesting that PLAT, LAMB2, and F9 may be independent predictors of DHF.

The upregulated proteins in groups A vs. C and B vs. C were highly enriched in the proteasome pathway. The proteasome degradation pathway is essential for many cell processes, including the cell cycle, oxidative stress response, and regulation of gene expression. Choy et al. (2015b) used RNAi technology to silence the components of the UP-proteasome in mosquitoes and observed a decrease in the production of infectious DENV virus. It is suggested that the proteasome pathway plays an important role in regulating the production of infectious DENV. The inhibition of ubiquitin-proteasome components and a transcriptomic analysis demonstrated that the ubiquitin-proteasome pathway is critical for DENV replication and transmission (Hicke, 2001; Chu and Ng, 2004; Rattiyaporn et al., 2010; Maria-Dolores et al., 2011; Choy et al., 2015a).

Alanine, aspartate, and glutamate metabolism and arginine biosynthesis were also the most significantly enriched pathways for the upregulated proteins. Amino acids are mainly building blocks for proteins. Enhancement of proteasome pathway activity increases amino acid metabolism. Amino acids also have other important functions. Studies by Engskog et al. (2017) showed that the alanine, aspartate, and glutamate metabolism pathway is critical for neurotransmission in neuroblastoma cells. Wang et al. (2012) found that the alanine, aspartate, and glutamate metabolic pathways in jaundice syndrome (JS) patients were affected. Mendes-Ribeiro et al. (2008) observed that when patients were infected with dengue virus, platelet count decreased and L-arginine NO pathway activity increased. In this study, we measured the clinical parameters of platelets. The mean platelet count in DF patients was lower than that in healthy controls, and DHF patients had the lowest number of platelets. Moreover, the upregulated proteins in groups A vs. C and B vs. C were enriched in the arginine biosynthesis pathway. This finding is consistent with Ribiero’s research.

Several upregulated proteins as markers related to the process of dengue virus infection were identified in this study. Similar to the results of Penelopie et al. (2004) and Murgue et al. (2001), VCAM1 was involved in the progress of dengue disease, and VCAM1 was significantly elevated in DSS patients. FGL1 belongs to the fibrinogen family and is associated with coagulation. FGL1 is also involved in mitosis and metabolism in liver cells (Zhilin and Chinweike, 2008; Demchev et al., 2013). The most recent research proves that FGL1 is involved in cellular immunity (Wang et al., 2019). MFAP4 is a glycoprotein related to elastic fibers and is located outside the cell. MFAP4 participates in the assembly of elastic fibers (Kasamatsu et al., 2011). MFAP4 could also be involved in calcium-dependent cell adhesion or intercellular interactions (Pilecki et al., 2016). Glutamine synthetase (GLUL) is an enzyme that catalyzes the conversion of glutamate to glutamine in a manner dependent on ATP (Johannes et al., 2005). GLUL functions in many biological processes, such as cellular signaling (Eisenberg et al., 2000; Youji et al., 2010; Natalia et al., 2015).

Three proteins were significantly elevated only in DHF, suggesting that these proteins may be independent predictors of DHF. Tissue-type plasminogen activator (PLAT) is an enzyme that converts plasminogen to plasmin. This enzyme plays an important role in biological processes such as tissue formation and cell migration. LAMB2, a laminin beta 2 subunit, contains 1798 amino acids. Laminin is a heterotrimeric extracellular glycoprotein composed of three subunits – α, β, and γ – that plays an important role in cell adhesion, proliferation, differentiation and migration. Maselli’s case study of congenital myasthenic syndrome (CMS) caused by mutations in the LAMB2 gene indicates that LAMB2 plays an important role in the development of neuromuscular junctions in humans (Maselli et al., 2009). Coagulation factor IX (gene name: F9) is a vitamin K-dependent plasma protein that participates in the intrinsic pathway of blood coagulation by converting coagulation factor X to its active form in the presence of Ca_2_ + ions, phospholipids, and coagulation factor VIII-a. Immunofluorescence staining of platelet coagulation factor V and coagulation factor IX was performed by Romp et al. (1993). Observation of the different states of the platelets (activated and non-activated) indicated that platelets contain coagulation factor IX and that coagulation factor IX is released at the active coagulation site.

These proteins as markers possibly revealed three mechanisms in the development of DF. One mechanism is vascular permeability. Increased vascular permeability leads to bleeding. A study by van Gorp et al. (1999) showed that the increase in the binding of VCAM1 and other adhesion molecules to leukocytes and platelets results in damage to endothelial cells, leading to bleeding. VCAM1, MFAP4, and LAMB2 are all associated with cell adhesion. We found that the levels of VCAM1 and MFAP4 in serum of DENV patients increased significantly in DF and increased even more in DHF; thus, these proteins could serve as markers for DHF prediction. In addition, LAMB2 increased only in DHF and could be used as a marker for DHF. Another mechanism is associated with hemorrhage/coagulation. FGL1 belongs to the family of fibrinogens, which are precursors of fibrin and act in the final stage of coagulation. PLAT is a plasminogen activator, and plasmin can degrade fibrin. An increase in plasminogen can cause an increased risk of bleeding. Coagulation factor IX is released at the site of bleeding and participates in coagulation by converting coagulation factor X to its active form. The expression of FGL1 increased to different degrees in DF and DHF/DSS. PLAT and F9 increased only in DHF/DSS. These molecules could be used as a marker for DHF/DSS prediction and a marker for DHF/DSS identification, respectively. The final mechanism is immune escape. Wang et al. (2019) generated FGL1 knockout transgenic mice. It was found that the immune activity of T cells in FGL1 knockout mice was enhanced, suggesting that FGL1 could inhibit the activation of antigen-specific T cells. Through their research on cancer, these researchers found that FGL1 is the main ligand of the immunosuppressive receptor LAG-3 and participates in the immune escape mechanism of tumor cells. In this study, FGL1 expression increased in DF and DHF/DSS to different degrees, suggesting that DENV virus may utilize the same immune escape mechanism as cancer cells.

In conclusion, using TMT-labeled proteomics, PLAT, LAMB2, F9, VCAM1, FGL1, MFAP4, and GLUL, which are related to the process of DENV infection were identified as predictors of DHF. These findings provide a reliable method for the diagnosis and classification of DF and DHF.

## Data Availability Statement

Publicly available datasets were analyzed in this study. This data can be found here: IPX0001691000.

## Ethics Statement

The studies involving human participants were reviewed and approved by the Mengchao Hepatobiliary Hospital of Fujian Medical University. The patients/participants provided their written informed consent to participate in this study.

## Author Contributions

LH, XA, and SL performed the experiments and wrote the draft manuscript. SG and LZ prepared the clinical samples. XH and HY designed the project, guided the experiments, and finally wrote the manuscript.

## Conflict of Interest

The authors declare that the research was conducted in the absence of any commercial or financial relationships that could be construed as a potential conflict of interest.

## Footnotes

1 http://www.UniProt.org/

2 http://imagej.nih.gov/ij/
