# Supplementary material for: Quantitative Comparative Proteomics Reveal Biomarkers for Dengue Disease Severity
Source: Front Microbiol. 2019 Dec 10;10:2836. doi: 10.3389/fmicb.2019.02836 (PMC6914681; doi:10.3389/fmicb.2019.02836)
Supplement: Supplementary file 1 [file Presentation_1.PPTX]

## Slide 1
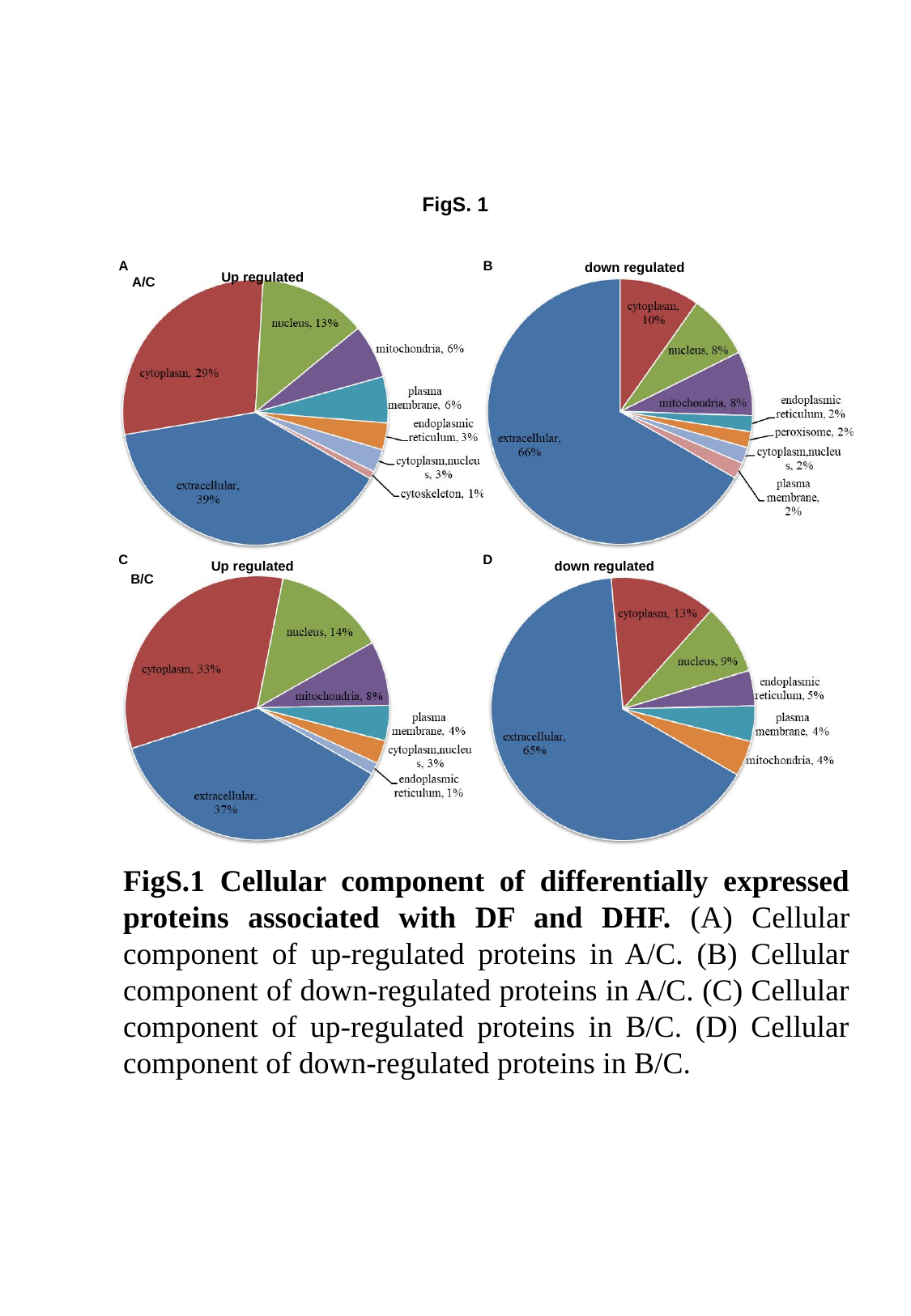

FigS. 1
A
B
down regulated
Up regulated
A/C
D
C
Up regulated
down regulated
B/C
FigS.1 Cellular component of differentially expressed proteins associated with DF and DHF. (A) Cellular component of up-regulated proteins in A/C. (B) Cellular component of down-regulated proteins in A/C. (C) Cellular component of up-regulated proteins in B/C. (D) Cellular component of down-regulated proteins in B/C.

## Slide 2
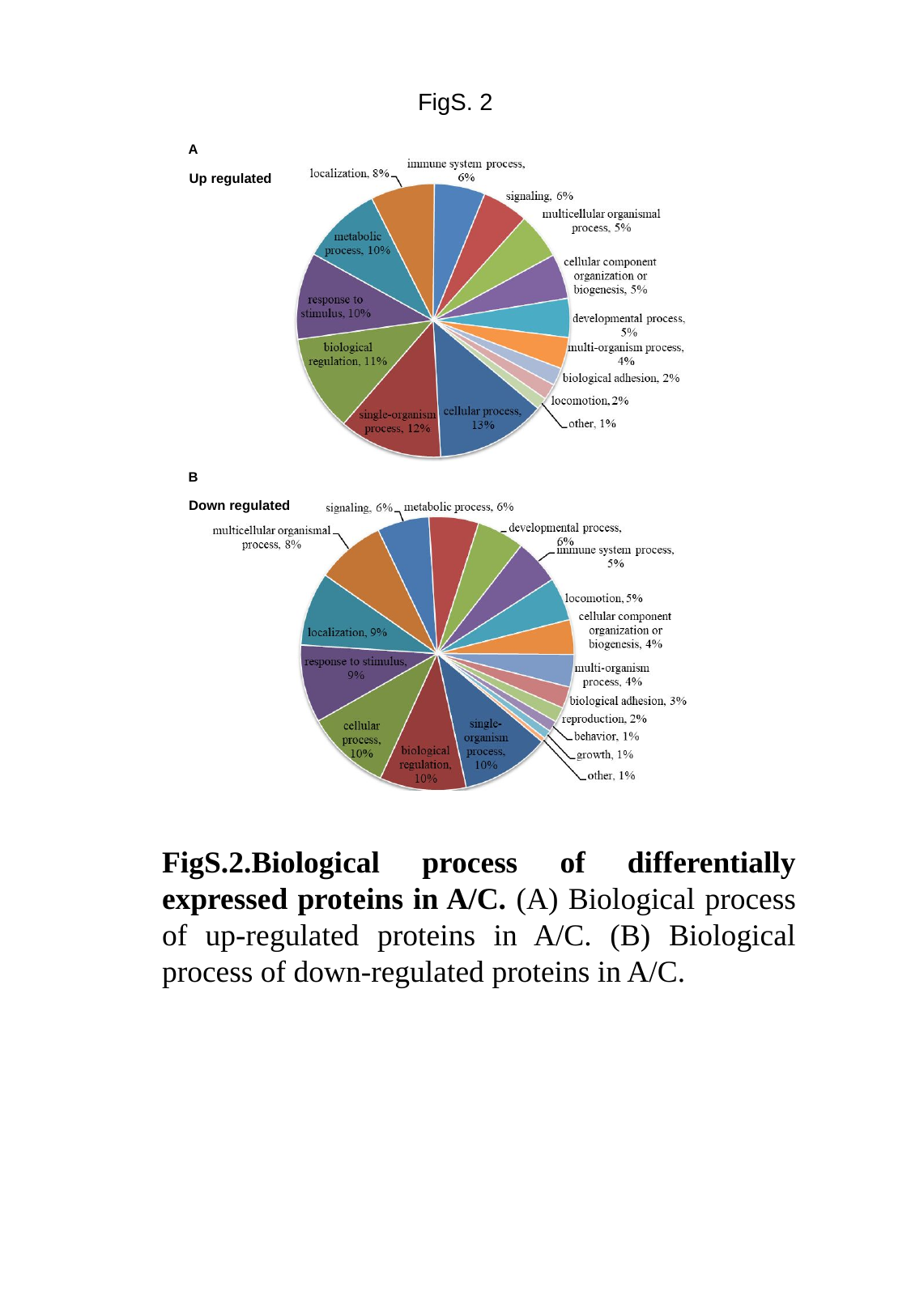

FigS. 2
A
Up regulated
B
Down regulated
FigS.2.Biological process of differentially expressed proteins in A/C. (A) Biological process of up-regulated proteins in A/C. (B) Biological process of down-regulated proteins in A/C.

## Slide 3
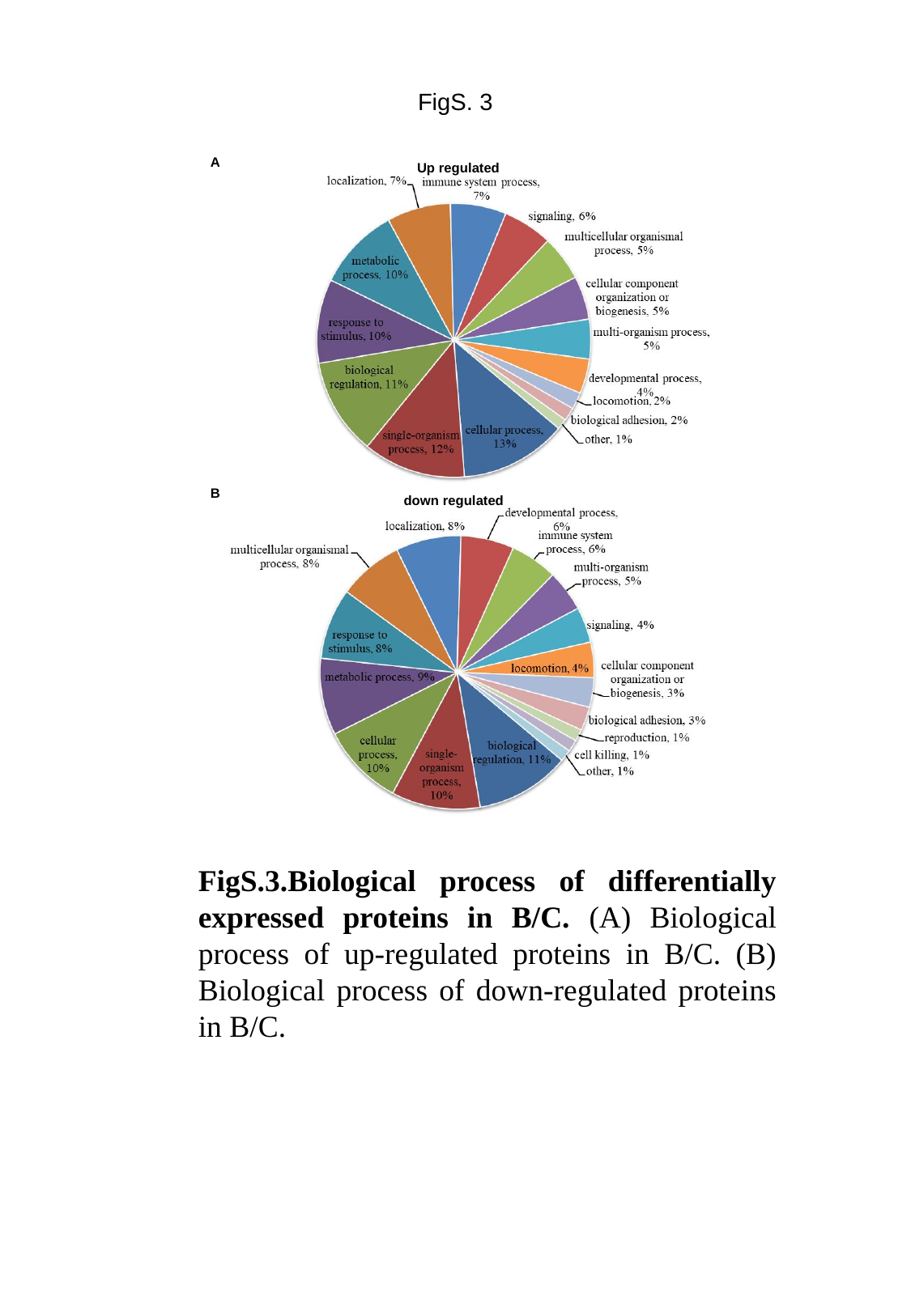

FigS. 3
A
Up regulated
down regulated
B
FigS.3.Biological process of differentially expressed proteins in B/C. (A) Biological process of up-regulated proteins in B/C. (B) Biological process of down-regulated proteins in B/C.
